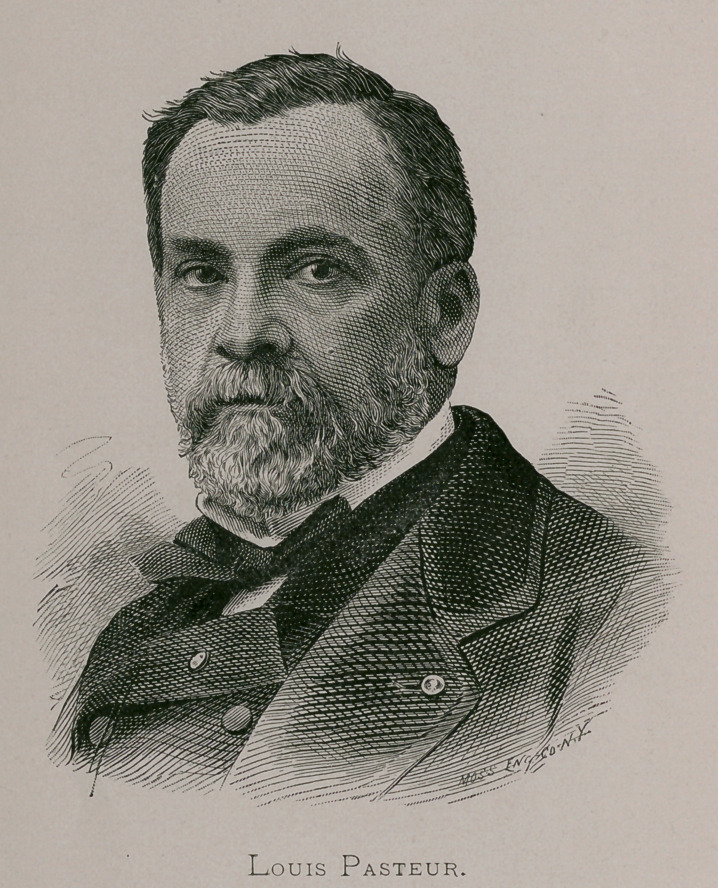# The Life Work of Pasteur*This “Review on the Life and Work of Pasteur” is taken from the *Popular Science Monthly* of June, 1884:We cordially recommend the careful reading of this book to every American veterinarian. The present movements in Preventive Medicine are especially dependant upon studies of certain animal diseases and as the principal work in this important field has been done by Pasteur, it is essential that all veterinarians, students especially, should be well acquainted with his life and work, which is very clearly detailed in the volume in question.

**Published:** 1885-01

**Authors:** 


Louis Pasteur.

### Art. VII.—THE LIFE-WORK OF PASTEUR.*

BY HIS SON-IN-LAW.

Louis Pasteur passed his childhood in a small tannery which his father had bought in the city of Arbois, in the department of the Jura, to which he removed from the ancient city of Dôle, in the same department, where he was born. When Louis became of suitable age, he was sent to the communal school, and was so proud of the fact that, though he was the smallest of the pupils, he went on the first day with his arms full of dictionaries away beyond his years. He does not appear, as yet, to have been a particularly diligent student. He was as likely to be found drawing a portrait or a sketch—and the walls of several Arboisian houses bear testimonies of his skill in this art—as studying his lesson, and to go a-hunting or a-fishing as to take the direct way to the school. Yet the principal of the college was ready to predict that it was no small school like this one, but some great royal institution, that was destined to enjoy his services as a professor. As there was no Professor of Philosophy in the college at Arbois, young Pasteur went to Besançon to continue his studies. Here, in the chemistry-class, he so vexed Professor Darlay with his frequent and searching questions, that the old gentleman was disconcerted, and declared it was his business to question the pupil, not Pasteur's to question him. Pasteur then had recourse to a pharmacist in the town who had gained some distinction in science, and took private lessons in chemistry from him. He fared better at the École Normale, where he had Balard for a teacher, and also enjoyed the instructions of

---

* This "Review on the Life and Work of Pasteur" is taken from the *Popular Science Monthly* of June, 1884:

We cordially recommend the careful reading of this book to every American veterinarian. The present movements in Preventive Medicine are especially dependant upon studies of certain animal diseases and as the principal work in this important field has been done by Pasteur, it is essential that all veterinarians, students especially, should be well acquainted with his life and work, which is very clearly detailed in the volume in question.

Dumas, with whom he formed a life-long friendship at the Sorbonne.

Pasteur's first important investigation was suggested at about this time, by an observation of Mitscherlich, the German mineralogist, of a difference in the behavior toward polarized light of the crystals of paratartrate of soda and ammonia and tartrate of soda and ammonia, bodies identical in composition and external form and other properties. Pasteur discovered differences in the form of the crystals and the molecular structure of the two bodies that had escaped detection, and was led to consider that all things may be divided into two categories: those having a plane of symmetry—that is, capable of being divided so that the parts on either side of the plane of division shall be equal and identical—or symmetrical bodies; and dissymmetrical bodies, or those not capable of being so divided. Occupied with the idea that symmetry or dissymmetry in the molecular arrangement of any chemical substance must be manifested in all its properties capable of showing the quality, he persued his investigations till he reached the conclusion that an essential difference in properties as to symmetry exists between mineral and dead matter and matter in which life is in course of development, the former being symmetrical, the latter unsymmetrical.

Pasteur's wedding-day came on while he was engaged in this investigation. He went, not to the marriage-feast, but to his laboratory, and had to be sent for when all was ready.

With his observing powers quickened by his studies of symmetry and dissymmetry, Pasteur went to the researches with which his life has been identified, beginning with his studies in fermentation. Liebig's theory, that fermentation is a change undergone by nitrogenous substances under the influence of the oxygen of the air, ruled at the time, and the observations of Schwann and Cagniard-Latour on the yeast-plant were overlooked or regarded as exceptional. M. Pasteur continued the investigation of the alcohol-producing yeast-plant, and, cultivating it in suitable solutions, proved that it possessed organizing power ample to account for the phenomena. He found a similar organism—minute cells or articulations narrowly contracted in the middle—active in the lactic fermentation, capable of cultivation; and another organism, a vibrion.

full of motion, living singly or in chains, working in the butyric fermentation.

The butyric vibrion was found to work quite as vigorously and with as much effect when no air was added to the decoctions, and in fact to perish with a stoppage of the formation of butyric acid when air was too freely supplied. Reverting to the development of the yeast-plant and the alcoholic fermentation, he found that they also went on best when free air was excluded. Thus, Liebig's dictum, that fermentation is the result of the action of oxygen, must be reversed or abandoned. The organisms working these processes were given the class-name of *anœorbes*, or beings that live without air. The French Academy's impressions of the results of Pasteur's work were spoken by Dumas, who said to him, "In the infinitely little of life you have discovered a third kingdom to which belong those beings which, with all the prerogatives of animal life, have no need of air to live, and find the heat they require in the chemical decompositions they provoke around them." The place of the organisms in the economy of Nature had not yet been fixed, but Pasteur was able to declare: "Whether the progress of science makes the vibrion a plant or an animal, is no matter; it is a living being endowed with motion, that lives without air and is ferment." It would be mere repetition to follow the experiments in putrefaction, where Liebig had denied that living organisms have any place, into which Pasteur carried the same methods and obtained the same results as in the case of fermentation. He proved that living organisms have all to do with it.

After M. Pasteur had been collecting his proofs for twenty years, Dr. Bouillaud sharply asked in the Academy: "How are your microscopic organisms disposed of? What are the ferments of the ferments?" He, as well as Liebig, believed the question could not be answered. Pasteur proved, by a series of the parallel experiments of the kind that have since become familiar, that oxygen deprived of its germs is incapable of producing fermentation or putrefaction, even after years, while the same substances are acted upon at once if the germs are present; and then answered that the ferments are destroyed by a new series of organisms—*œrobes*—living in the air, and these by other ærobes in succession, until the ultimate products are oxidized. "Thus, in the destruction of what has lived, all is reduced to the simultaneous action of the three great natural phenomena—fermentation, putrefaction and slow combustion. A living being, animal or plant, or the *débris* of either, having just died, is exposed to the air. The life that has abandoned it is succeeded by life under other forms. In the superficial parts accessible to the air, the germs of the infinitely little ærobes flourish and multiply. The carbon, hydrogen and nitrogen of the organic matter are transformed, by the oxygen of the air and under the vital activity of the ærobes, into carbonic acid, the vapor of water and ammonia. The combustion continues as long as organic matter and air are present together. At the same time the superficial combustion is going on, fermentation and putrefaction are performing their work in the midst of the mass by means of the developed germs of the anærobes, which not only do not need oxygen to live, but which oxygen causes to perish. Gradually the phenomena of destruction are at last accomplished through the work of latent fermentation and slow combustion. Whatever animal or vegetable matter is in the open air or under the ground, which is always more or less impregnated with air, finally disappears. The processes can be stopped only under an extremely low temperature, . . . . in which the microscopic organisms cannot flourish. These facts come in to fortify the still new ideas of the part which the infinitely little play as masters of the world. If their work, always latent, were suppressed, the surface of the globe, overloaded with organic matters, would become uninhabitable."

Pasteur extended his observations to the acetic fermentation or conversion of alcohol into vinegar, in which he found an organism, the *Mycoderma aceti*, actively promoting a process of oxidation. Liebig had attributed this fermentation, also, to the presence of an albuminoid body in process of alteration, and capable of fixing oxygen. He knew of the plant called "mother," but regarded it as an outgrowth of the fermentation, and in no sense the cause. Pasteur proved by experiments that left no room for doubt—the prominent characteristic feature in all his investigations—that the plant is the real agent in producing the fermentation. He eliminated from his compositions the albuminoid matter, which Liebig had declared to be the active agent, and replaced it with crystallizable salts, alkaline phosphates, and earths; then, having added alcoholized water, slightly acidulated with acetic acid, he saw the mycoderm develop, and the alcohol change into vinegar. Having tried his experiments in the vinegar factories at Orleans, he became so sure of his position that he offered to the Academy, in one of its discussions, to cover with the mycoderm, within twenty-four hours, from a few hardly visible sowings, a surface of vinous liquid as entensive as the hall in which they were meeting.

Liebig allowed ten years to pass after Pasteur's investigations, and then published a long memoir traversing his conclusions. Pasteur visited Liebig at Munich in 1870, to discuss the matter with him. The German chemist received him courteously, but excused himself from the discussion on the ground of a recent illness. The Franco-German War came on, but as soon as it was over Pasteur invited Liebig to choose a committee of the Academy and furnish a sugared mineral liquid. He would produce in it, before them all, an alcoholic fermentation in such a way as to establish his own theory and contradict Liebig's. Liebig had referred to the process of preparing vinegar by passing diluted alcohol through wooden chips, as one in which no trace of a mycoderm could be found, but in which the chips appeared perfectly clean after each operation. It was, in fact, impossible that there should be any mycoderm, because there was nothing on which it could be fed. Pasteur replied to this: "You do not take account of the character of the water with which the alcohol is diluted. Like all common waters, even the purest, it contains ammoniacal salts and mineral matters that can feed the plants, as I have already demonstrated. You have, moreover, not carefully examined the surface of the chips with the microscope. If you had, you would have seen the little articles of the *Mycoderma aceti*, sometimes joined into an extremely thin pellicle that may be lifted off. If you will send me some chips from the factory at Munich, selected by yourself in the presence of its director, I will, after drying them quickly in a stove, show the mycoderm on their surface to a committee of the Academy charged with the determination of this debate." Liebig did not accept the challenge, but the question involved has been decided.

The experiments in fermentation led by natural steps to the debate on spontaneous generation, in which Pasteur was destined to settle a question that had interested men ever since they lived. The theory that life originates spontaneously from dead matter had strong advocates, among the most earnest of whom was M. Pouchet. He made a very clear presentment of the question at issue, saying: "The adversaries of spontaneous generation assume that the germs of microscopic beings exist in the air and are carried by it to considerable distances. Well! what will they say if I succeed in producing a generation of organized beings after an artificial air has been substituted for that of the atmosphere?" Then he proceeded with an experiment in which all his materials and vessels seemed to have been cleansed of all germs that might possibly have existed in them. In eight days a mold appeared in the infusion, which had been put boiling-hot into the boiling-hot medium. "Where did the mold come from," asked M. Pouchet, triumphantly, "if it was not spontaneously developed?" "Yes," said M. Pasteur, in the presence of an enthusiastic audience, for Paris had become greatly excited on the subject, "the experiment has been performed in an irreproachable manner as to all the points that have attracted the attention of the author; but I will show that there is one cause of error that M. Pouchet has not perceived, that he has not thought of, and no one else has thought of, which makes his experiment wholly illusory. He used mercury in his tub, without purifying it, and I will show that that was capable of collecting dust from the air and introducing it into his apparatus." Then he let a beam of light into the darkened room, and showed the air full of floating dust. He showed that the mercury had been exposed to atmospheric dust ever since it came from the mine, and was so impregnated and covered with it as to be liable to soil everything with which it came in contact. He instituted experiments similar to those of M. Pouchet, but with all the causes of error that had escaped him removed, and no life appeared. The debate, which continued through many months, and was diversified by a variety of experiments and counter-experiments, was marked by a number of dramatic passages and drew the attention of the world. M. Pasteur detected a flaw in every one of M. Pouchet's successful experiments, and followed each one with a more exact experiment of his own, which was a triumph for his position. Having shown by means of bottles of air collected from different heights in a mountain region, that the number of germs in the air diminishes with the elevation above the earth, and that air can be got free from germs and unproductive, M. Pasteur asserted decisively: "There is no circumstance now known that permits us to affirm that microscopic beings have come into the world without germs, without parents like themselves. Those who affirm it have been victims of illusions, of experiments badly made, and infected with errors which they have not been able to perceive or avoid. Spontaneous generation is a chimera." M. Flourens, Perpetual Secretary of the Academy, said: "The experiments are decisive. To have animalcules, what is necessary if spontaneous generation is real? Air and putrescible liquids. Now, M. Pasteur brings air and putrescible liquids together, and nothing comes of it. Spontaneous generation, then is not. To doubt still is not to comprehend the question." There were, however, some who still doubted, and to satisfy them M. Pasteur offered, as a final test, to show that it was possible to secure, at any point, a bottle of air containing no germs, which would, consequently, give no life. The Academy's committee approved the proposition; but M. Pouchet and his friends pleaded for delay, and finally retired from the contest.

The silk-raising industry of the South of France was threatened with ruin by a disease that was destroying the silk-worms, killing them in the egg, or at a later stage of growth. Eggs, free from the disease, were imported from other countries. The first brood flourished, but the next ones usually fell victims to the infection, and the malady spread. All usual efforts to prevent it or detect its cause having failed, a commission was appointed to make special investigations, and M. Pasteur was asked to direct them personally. He did not wish to undertake the work, because it would withdraw him from his studies of the ferments. He, moreover, had never had anything to do with silk-worms. "So much the better," said Dumas. "You know nothing about the matter, and have no ideas to interfere with those which your observations will suggest." Theories were abundant, but the most recent and best authorities agreed that the diseased worms were beset by corpuscles, visible only under the microscope. He began his investigations with the idea that these corpuscles were connected with the disease, although assurances were not wanting that they also existed in a normal condition of the silk-worm. M. Pasteur's wife and daughters, and his assistants in the normal school, associated themselves with him in the studies, and became, for the time, amateur silk-raisers. He studied the worms in every condition, and the corpuscles in every relation, for five years. He found that there were two diseases—the contagious, deadly *pébrine*, the work of the corpuscles, and *flachery*, produced by an internal organism ; and "became so well acquainted with the causes of the trouble and their different manifestations that he could, at will, give *pébrine* or *flachery*. He became able to graduate the intensity of the disease, and make it appear at any day and almost at any hour." He found the means of preventing the disorders, and "restored its wealth to the desolated silk district." The cost of this precious result was a paralysis of the left side, from which he has never fully recovered.

As early as 1860 M. Pasteur expressed the hope that he might "be able to pursue his investigations far enough to prepare the way for a more profound study of the origin of diseases." Reviewing, at the conclusion of his "Studies on Beer," the principles which had directed his labors for twenty years, he wrote that the etiology of contagious diseases was, perhaps, on the eve of receiving an unexpected light. Robert Boyle had said that thorough understanding of the nature of fermentations and ferments might give the key to the explanation of many morbid phenomena. The German doctor, Traube, had in 1864 explained the ammoniacal fermentation of urine, by reference to Pasteur's theory. The English surgeon, Dr. Lister, wrote in 1874 to Pasteur that he owed to him the idea of the antiseptic treatment of wounds which he had been practicing since 1865. Professor Tyndall wrote to him, in 1876, after having read his investigations for the second time : "For the first time in the history of science we have a right to entertain the sure and certain hope that, as to epidemic diseases, medicine will shortly be delivered from empiricism and placed upon a really scientific basis. When that great day shall come, mankind will, in my opinion, recognize that it is to you that the greatest part of its gratitude is due."

The domestic animals of France and other countries had been subject to a carbuncular disease, like the malignant pustule of man, which took different forms and had different names in different species, but was evidently the same in nature. A medical commission had, between 1849 and 1852, made an investigation of it and found it transmissible by inoculation from animal to animal. Dr. Davaine and Rayer had, at the same time, found in the blood of the diseased animals minute filiform bodies, to which they paid no further attention for thirteen years, or till after Pasteur's observations on fermentation had been widely spread. Then, Davaine concluded that these corpuscles were the source of the disease. He was contradicted by MM. Jaillard and Leplat, who had inoculated various animals with matter procured from sheep and cows that had died of the disease without obtaining a development of the bodies in question. Davaine suggested that they had used the wrong matter, but they replied that they had obtained it direct from an unmistakable source. Their views were supported by the German Dr. Koch and M. Paul Bert. At this point, M. Pasteur stepped in and began experiments after methods which had served him as sure guides in his studies of twenty years. They were at once simple and delicate. "Did he wish, for example, to demonstrate that the microbe-ferment of the butyric fermentation was also the agent in decomposition? He would prepare an artificial liquid, consisting of phosphate of potash, magnesia, and sulphate of ammonia, added to the solution of fermentable matter, and in the medium thus formed would develop the microbe-ferment from a pure sowing of it. The microbe would multiply and provoke fermentation. From this liquid he would pass to a second and then to a third fermentable solution of the same composition, and so on, and would find the butyric fermentation appearing in each successively. This method had been sovereign in his studies since 1857. He now proposed to isolate the microbe of blood infected with carbuncle, cultivate it in a pure state, and study its action on animals." As he was still suffering from a partial paralysis, he employed M. Joubert to assist him and share his honors. In April, 1877, he claimed before the Academy of

Sciences that he had demonstrated, beyond the possibility of a reply, that the bacillus discovered by Davaine and Rayer in 1850 was in fact the only agent in producing the disease. It still remained to reconcile the facts adduced by Messrs. Jaillard and Leplat with this assertion. The animals which they had inoculated died, but no bacteria could be found in them. M. Paul Bert, in similar experiments, had found a disease to persist after all bacteria had been destroyed. An explanation of the discrepancy was soon found.

The bacteria of carbuncle are destroyed as soon as putrefaction sets in. The virus with which these gentlemen had experimented was taken from animals that had been dead twenty-four hours and had begun to putrefy. They had inoculated with putrefaction, and produced septicæmia instead of carbuncle. All the steps in this line of argument were established by irrefragable proof. M. Pasteur afterward had a similar controversy with some physicians of Turin, at the end of which they shrank from the test experiment he offered to go and make before them. "Remember," shortly afterward said a member of the Academy of Sciences to a member of the Academy of Medicine, who was going—in a scientific sense—to "choke" M. Pasteur, "M. Pasteur is never mistaken."

Having discovered and cultivated the microbe that produces hen-cholera, Pasteur turned his attention to the inquiry whether it would be possible to apply a vaccination to the prevention of these terrible diseases of domestic animals. He found that he could transplant the microbe of hen-cholera to an artificially prepared medium and cultivate it there, and transplant it and cultivate it again and again, to the hundredth or even the thousandth time, and it would retain its full strength—provided too long an interval was not allowed to elapse between the successive transplantations and cultures. But if several days or weeks or months passed without a renewal of the medium, the culture being all the time exposed to the action of oxygen, the infection gradually lost in intensity. A virus was produced of a strength that would make sick, but not kill. Hens were inoculated with this, and then, after having recovered from its effects, with virus of full power. It made them sick, but they recovered. A preventive of hen-cholera had been found. In the experiments upon the feasibility of applying a similar remedy to carbuncular diseases, it was necessary to ascertain whether or not animals, which had once been stricken with the disease,were exempt from liability to a second attack. The investigator was met at once by the formidable difficulty that no animals were known to have recovered from a first attack, to serve as subjects for trial. A fortunate accident in the failure of another investigator's experiment gave M. Pasteur a few cows that had survived the disease. They were inoculated with virus of the strongest intensity, and were not affected. It was demonstrated, then, that the disease would not return. M. Pasteur now cultivated an attenuated carbuncle-virus, and, having satisfied himself that vaccination with it was effective, declared himself ready for a public test-experiment. Announcing his success to his friends, he exclaimed in patriotic self-forgetfulness, "I should never have been able to console myself, if such a discovery as I and my assistants have just made had not been a French discovery!"

Twenty-four sheep, a goat and six cows were vaccinated, while twenty-five sheep and four cows were held in reserve, unvaccinated, for further experiment. After time had been given for the vaccination to produce its effect, all of the animals, sixty in number, were inoculated with undiluted virus. Forty-eight hours afterward more than two hundred persons met in the pasture to witness the effect. Twenty-one of the unvaccinated sheep and the goat were dead, and two more of the sheep were dying, while the last one died the same evening; the unvaccinated cows were suffering severely from fever and œdema. The vaccinated sheep were all well and lively, and the vaccinated cows had neither tumor nor fever of any kind, and were feeding quietly. Vaccination is now employed regularly in French pastures; five hundred thousand cases of its application had been registered at the end of 1883; and the mortality from carbuncle has been reduced ten times.

There is no need to follow M. Pasteur in his further researches in the *rouget* of pork, in boils, in puerperal fever, in all of which, with other maladies, he has applied the same methods with the same exactness that have characterized all his work. His laboratory at the École Normale is a collection of animals to be experimented upon—mice, rabbits, Guineapigs, pigeons, and other suitable subjects, with the dogs upon which he is now studying hydrophobia most prominent. There is nothing cruel in his work. His inoculations are painless, except as the sickness they induce is a pain, and the suffering they cause is as nothing compared with that which they are destined to save. On this subject he himself has remarked in one of his lectures: "I could never have courage to kill a bird in hunting; but, in making experiments I have no such scruples. Science has a right to invoke the sovereignty of the end."

What he has done, M. Pasteur regards as only the beginning of what is to be accomplished in the same line. "You will see," he has sometimes said, "how this will grow as it goes on. Oh, if I only had time!"

## Figures and Tables

**Figure f1:**